# Characterization of Novel Dipeptidyl Peptidase-IV Inhibitory Peptides from Soft-Shelled Turtle Yolk Hydrolysate Using Orthogonal Bioassay-Guided Fractionations Coupled with In Vitro and In Silico Study

**DOI:** 10.3390/ph13100308

**Published:** 2020-10-14

**Authors:** Nhung Thi Phuong Nong, Yu-Kuo Chen, Wen-Ling Shih, Jue-Liang Hsu

**Affiliations:** 1 Department of Tropical Agriculture and International Cooperation, National Pingtung University of Science and Technology, Pingtung 91201, Taiwan; npnhung91@gmail.com; 2 Department of Basic Science, Thainguyen University of Agriculture and Forestry, Quyetthang Ward, Thai Nguyen 250000, Vietnam; 3 Department of Food Science, National Pingtung University of Science and Technology, Pingtung 91201, Taiwan; chenyk@mail.npust.edu.tw; 4 Department of Biological Science and Technology, National Pingtung University of Science and Technology, Pingtung 91201, Taiwan; wlshih@mail.npust.edu.tw; 5 International Master’s Degree Program in Food Science, National Pingtung University of Science and Technology, Pingtung 91201, Taiwan; 6 Research Center for Animal Biologics, National Pingtung University of Science and Technology, Pingtung 91201, Taiwan

**Keywords:** DPP-IV inhibitory peptides, soft-shelled turtle yolk protein, bioassay-guided fractionation, LC-MS/MS, in silico analysis

## Abstract

Five novel peptides (LPLF, WLQL, LPSW, VPGLAL, and LVGLPL) bearing dipeptidyl peptidase IV (DPP-IV) inhibitory activities were identified from the gastrointestinal enzymatic hydrolysate of soft-shelled turtle yolk (SSTY) proteins. Peptides were isolated separately using reversed-phase (RP) chromatography in parallel with off-line strong cation exchange (SCX) chromatography followed by liquid chromatography-tandem mass spectrometry (LC-MS/MS) analysis to determine sequences. Among these peptides, LPSW showed the highest DPP-IV inhibitory activity with an IC_50_ value of 269.7 ± 15.91 µM. The results of the pre-incubation experiment and the kinetic study of these peptides indicated that WLQL is a true inhibitor and its inhibition toward DPP-IV is of an uncompetitive model, while LPLF, LPSW, and VPGLAL are real-substrates and competitive inhibitors against DPP-IV. The DPP-IV inhibitory peptides derived from SSTY hydrolysate in study are promising in the management of hyperglycemia in Type 2 diabetes.

## 1. Introduction

The egg of soft-shelled turtle (SST) (*Pelodiscus sinensis*), a valuable aquaculture product in Taiwan and other Asian countries, is widely consumed as a tonic food in traditional Chinese medicine [1]. The soft-shelled turtle egg has been utilized for its biofunctional benefits, such as cholesterol-lowering effects and antihypertensive activities [2,3,4,5,6]. Cheung and Li reported that diabetes and hypertension share a common metabolic pathway, leading the therapeutic target to significantly reduce blood pressure and blood glucose levels [7]. Hydrolyzed soft-shelled turtle yolk (SSTY) proteins may be utilized in the control of type 2 diabetes via their capacity to inhibit DPP-IV. The present study focuses on using SSTY as a bioactive peptide source. Bioactive peptides contain few amino acids that are inactive in the parent protein [8]. However, these peptides exhibit a positive health impact on the human body after proteolytic digestion [9,10]. For this reason, they should be degraded from parent protein using enzymatic hydrolysis, microbial fermentation, gastrointestinal digestion, or other physical conditions [11,12].

Dipeptidyl peptidase (DPP-IV, EC 3.4.14.5) is a serine protease that cleaves preferentially dipeptides with the sequence X-proline and X-alanine from the N-terminus of a polypeptide. The enzyme DPP-IV is a ubiquitous enzyme expressed in the surface of various cells, particularly in the circulation, including the liver, kidney, and intestine. The functional enzymes can degrade incretins including glucagon-like-peptide-1 (GLP-1) and glucose-dependent insulinotropic polypeptide (GIP) [13]. Those incretins can stimulate insulin secretion from pancreatic beta cells in a glucose-dependent manner. The inactivation of GLP-1 or GIP by DPP-IV leads to the deficient insulin secretion that causes hyperglycemia, commonly found in type 2 diabetes. DPP-IV inhibitors are utilized for the preservation of the insulinotropic effect of the incretin, thereby increasing their half-life. The principle of using DPP-IV inhibitors as therapy for diabetes has been firmly established through the use of commercial synthetic medications such as sitagliptin, vildagliptin, saxagliptin, linagliptin, and alogliptin [14,15]. However, synthetic DPP-IV inhibitory drugs may present side effects such as pancreatitis, infective disorders, and angioedema [15,16]. Numerous research studies have emphasized the probability of utilizing food-derived proteins and peptides as a natural DPP-IV inhibitor source that may be safer than commercial drugs [17,18]. Dietary proteins such as milk [19,20], eggs [21,22], salmon [23], and rice bran [24] may be excellent sources of biologically active peptides.

This study aimed to screen the DPP-IV inhibitory peptides from SSTY hydrolysates selected from several enzymatic digests using two independent bioassay-guided fractionations; strong cation-exchange (SCX) chromatography and reversed-phase high-performance liquid chromatography (RP-HPLC). The bioactive peptides from SSTY hydrolysate were identified using liquid chromatography-tandem mass spectrometry (LC-MS/MS) coupled with database-assisted peptide sequencing. The BIOPEP database was also applied as an in silico analytical tool to predict the peptides’ DPP-IV inhibitory activities. The peptides’ IC_50_ values were further determined using synthetic peptides with corresponding sequences. To study their molecular mechanisms, the peptides’ inhibition kinetics on DPP-IV was further investigated using the Lineweaver–Burk plot. Additionally, the potent DPP-IV inhibitory peptides’ stabilities were examined upon DPP-IV hydrolysis using pre-incubation experiments, and the resulting fragments were monitored using LC-MS/MS.

## 2. Results and Discussion

### 2.1. Effects of Different Proteases on Hydrolysates’ DPP-IV Activities

**Figure 1:**
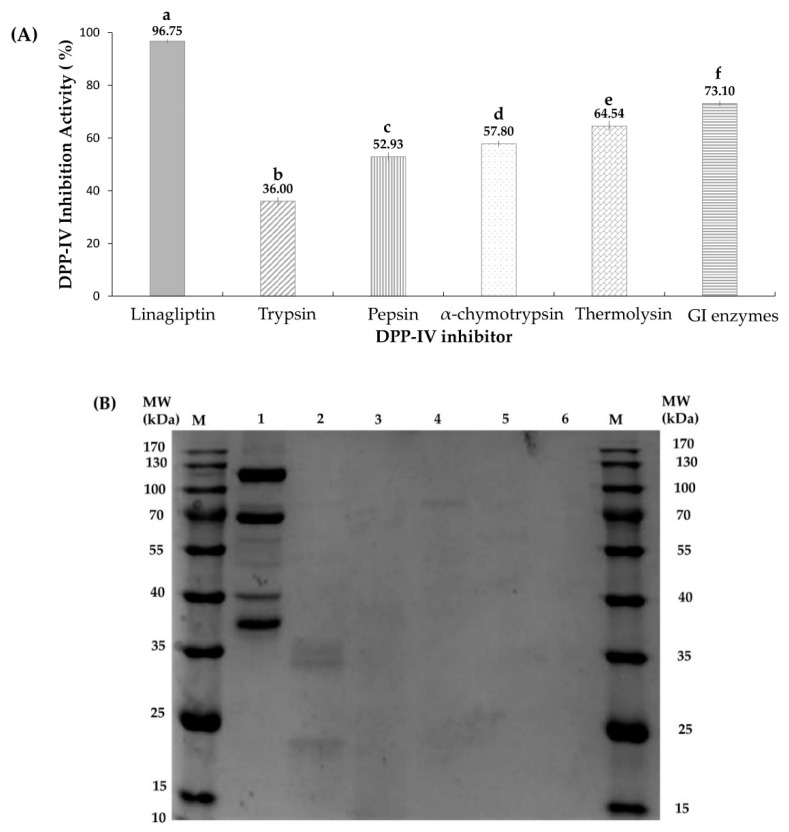
(**A**) DPP-IV inhibitory activity with soft shelled turtle yolk (SSTY) hydrolysates by trypsin, pepsin, α-chymotrypsin, and GI (pepsin, trypsin and α-chymotrypsin) enzyme, respectively. Different letters (a–f) indicate significant differences between samples (*p* < 0.05). (**B**) SDS-PAGE analysis of SSTY protein (Lane 1), trypsin digest (Lane 2), pepsin digest (Lane 3), α-chymotrypsin digest (Lane 4), thermolysin digest (Lane 5), and GI hydrolysate (Lane 6) of SSTY proteins at concentration 50 µg/µL. M represents the molecular weight of the protein markers.

In the present study, only short peptides (<3 kDa) were utilized to investigate DPP-IV inhibitory activities. Generally, smaller inhibitors are absorbed better into intestines, are more accessible toward the active sites of DPP-IV, and are easier to fractionate in the absence of larger peptides. Besides, lipids were eliminated from the SSTY protein to optimize enzymatic digestion activities and for further LC-MS/MS analysis. A DPP-IV inhibitory assay was carried out using the same concentration (2 mg/mL) of digest hydrolyzed by different proteases to select the best DPP-IV inhibitory hydrolysate. The positive control, Linagliptin (50 nM), showed the highest DPP-IV activity (96.75%). The GI (gastrointestinal enzymes containing pepsin, trypsin, and α-chymotrypsin) hydrolysate exhibited significantly higher DPP-IV inhibitory activity (73.10%) than those of other hydrolysates generated by trypsin (36.00%), pepsin (52.93%), α-chymotrypsin (57.80%), and thermolysin (64.54%) (Figure 1A). The SSTY proteins’ distribution and the hydrolysis degrees of hydrolysates produced by different enzymes were monitored by SDS-PAGE, as shown in Figure 1B. Four major protein bands of SSTY were observed and their identities were further characterized in Section 2.4. The SDS-PAGE also showed that trace of large peptides can be found in the hydrolysates of individual enzymes; trypsin, pepsin, α-chymotrypsin, and thermolysin (Lane 2–5 in Figure 1B). No detectable large peptides (>10 kDa) were found in GI hydrolysate (Line 6 in Figure 1B), which revealed that GI enzymes showed the best hydrolysis degree than those derived from other enzymes. GI enzymes preferentially produce peptides containing C-terminal hydrophobic residues (such as Phe, Tyr, Trp, and Leu) and alkaline residues (Lys and Arg). Therefore, the SSTY peptides resulting from GI hydrolysis are considered to be of smaller size and contain more hydrophobic or basic residues that intensify the interaction with the active site of DPP-IV [25].

The IC_50_ value of GI hydrolysate against DPP-IV was calculated based on the nonlinear regression of DPP-IV inhibition percentages (%) versus the logarithms of six concentrations of GI hydrolysate (<3 kDa). The IC_50_ value obtained was of 1.017 ± 0.0356 mg/mL. According to Nongonierma et al. [25], potent IC_50_ values against DPP-IV for most hydrolysate generated by gastrointestinal enzymes were around 1 mg/mL, which implies that the gastrointestinal hydrolysate of SSTY protein may contain potent DPP-IV inhibitory peptides.

### 2.2. Two Orthogonal Bioassay-Guided Fractionations of SSTY Gastrointestinal Hydrolysate

**Figure 2:**
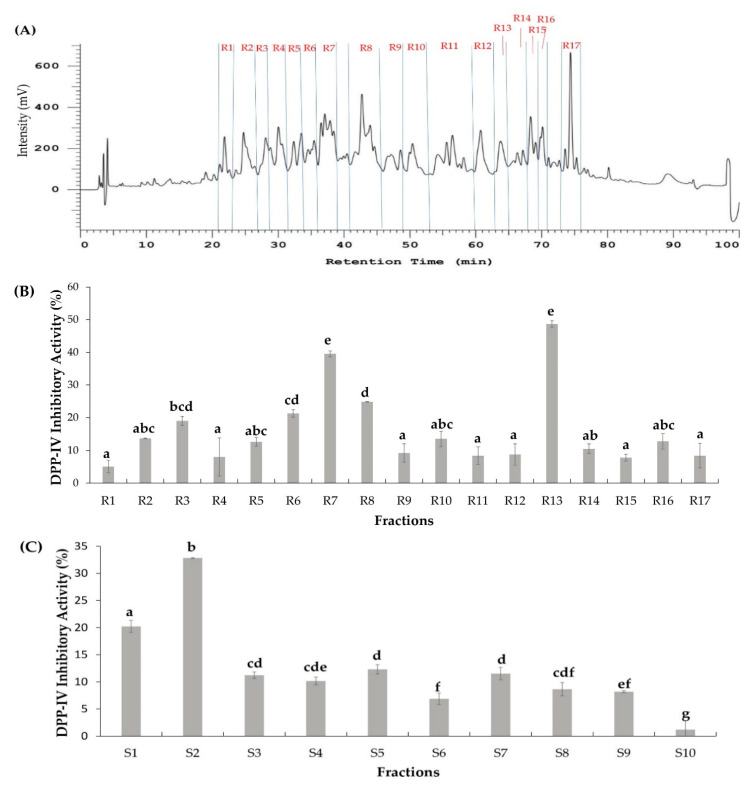
(**A**) Reverse-phase HPLC chromatogram with 10 mm × 250 mm, 5 µm column of turtle yolk hydrolysate. (**B**) DPP IV—Inhibition activity of RP-HPLC fractions (R1–R17). (**C**) DPP-IV inhibition activity of SCX fractions (S1–S10). Different letters (a–g) indicate significant differences between samples (*p* < 0.05).

Generally, peptides coexist in a mixture of active enzymatic hydrolysates. Therefore, a vital step in studying bioactive peptides is to know how to make them more efficiently accessible, thereby reducing researching time and finances to screen and eliminate inferior peptides. According to the different characters of each peptide such as the molecular weight, hydrophobicity, and other physicochemical properties, several orthogonal bioassay-guided methods were applied individually as well as the combination of two or three chromatography techniques which were utilized [4,26,27,28,29]. To fractionate SSTY GI hydrolysates, two column chromatography methods (RP and SCX) with different separation mechanisms were performed in parallel. RP-HPLC principle of separation depends on the difference in hydrophobic and hydrophilic properties of amino acids, while SCX is based on the anion and cationic components in the peptide. Therefore, the peptide mixture was separated to give different distribution orders. The peptides in the fractions with the highest DPP-IV inhibitory activities from both RP and SCX separations were identified using LC-MS/MS. The identical peptides identified from both fractions were regarded as the most potent DPP-IV inhibitory peptides [4]. In the RP separation technique, with the C18 column coupled with a UV-vis detector at 214 nm, 17 fractionations (R1−R17) were individually collected after every high peak (intensity > 150 mV) during 100 min (Figure 2A). After that, the DPP-IV inhibitory activity of each fraction at the same concentration (1 mg/mL) was examined. The result indicated that fraction R7 (RP-R7) and R13 (RP-R13) showed higher DPP-IV inhibitory activities (39.50% and 48.67%, respectively) compared with the other remaining RP fractions (Figure 2B).

Similarly, SSTY hydrolysate (<3 kDa) was fractionated using SCX chromatography. Among the 10 SCX fractions (S1−S10) produced by increasing salt concentrations of the mobile phase, the highest DPP-IV inhibitory activity (32.81%) was found in fraction S2 (SCX-S2) (Figure 2C).

### 2.3. Database-Assisted Peptide Sequencing and In Silico Prediction of Potential DPP-IV Inhibitory Peptides

**Figure 3:**
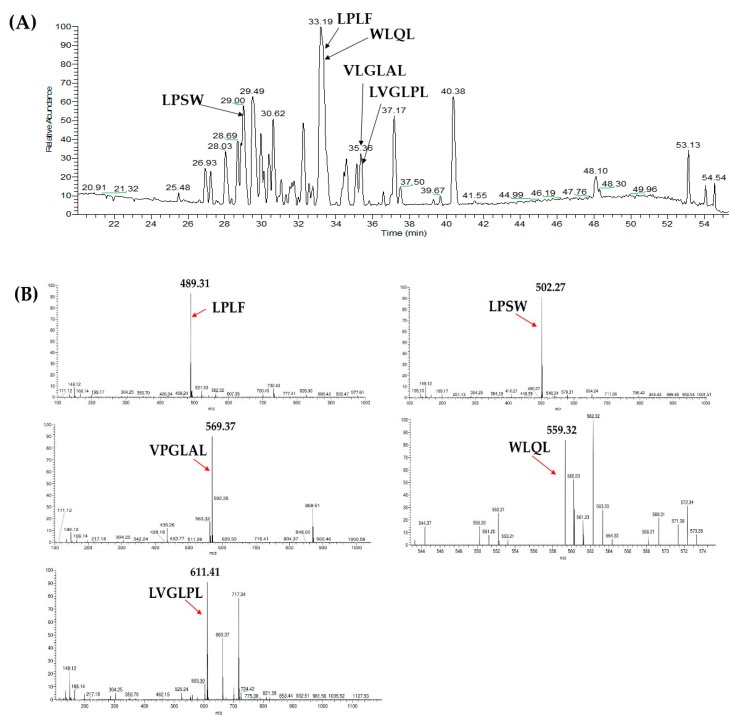

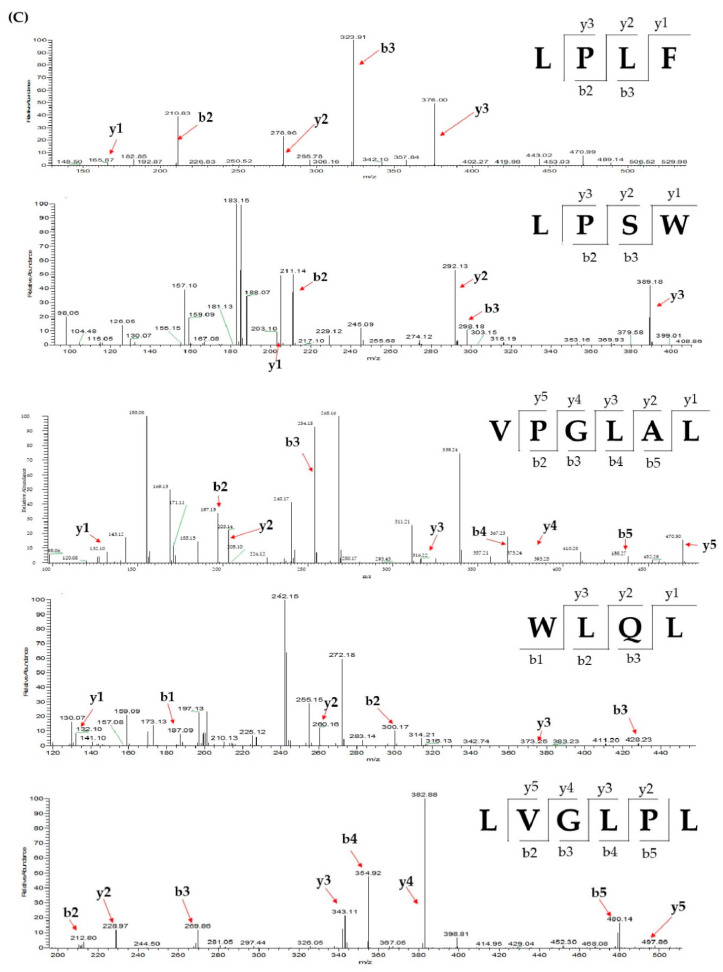
(**A**) The distribution of five overlapping peptides in LC-MS chromatogram of SCX-S2 fraction; (**B**) MS spectra of LPLF (*m*/*z* 489.31 at t_R_ = 32.31 min), LPSW (*m*/*z* 502.27 at t_R_ = 29 min), VPGLAL (*m*/*z* 569.37 at t_R_ = 30.31 min), WLQL (*m*/*z* 559.32 at t_R_ = 33.05 min), and LVGLPL (*m*/*z* 611.41 at t_R_ = 35.30 min); (**C**) MS/MS spectra of the peptide with *m*/*z* 489.31, 502.27, 569.37, 559.32, and 611.41, respectively. The b- and y-series ions in the MS/MS spectra indicate the fragment ions of the peptides.

The peptide sequences in fractions R7 and R13 of RP separation and fraction S2 of SCX separation were identified by LC-MS/MS analysis coupled with a database-assisted sequencing using the protein database of *P. sinensis* downloaded from NCBI. Twenty-one peptides were identified from fraction SCX-S2; while seven and nine peptides were identified from the RP-R7 and RP-R13 fractions, respectively. The information of identified peptides from SCX-S2, RP-R7, and RP-R13 fractions was summarized on Table 1. Two peptides including VPGLAL and LPSW were identically identified from SCX-S2 and RP-R7; while three identical peptides (LPLF, LVGLPL, and WLQL) were also characterized from SCX-S2 and RP-R13 (Table 1). The distribution of these five overlapping peptides in LC-MS chromatogram of SCX-S2 fraction was indicated on Figure 3A. Figure 3B shows the MS spectra of these five peptides, where the precursor ion exhibited *m*/*z* of 489.31 for LPLF (t_R_ = 32.31 min), 502.27 for LPSW (t_R_ = 29 min), 569.37 for VPGLAL (t_R_ = 30.31 min), 559.32 for WLQL (t_R_ = 33.05 min), and 611.41 for VPGLPL (t_R_= 35.30 min). The MS/MS spectra of these peptides, as well as their assignment of b- and y-series ions were illustrated in Figure 3C. These peptides’ identities were further confirmed using synthetic peptides with corresponding sequences by the comparison of their *m*/*z* values, retention times, and MS/MS spectra.

To predict the biological activities and toxicities of these peptides, BIOPEP and ToxinPred were performed. According to the prediction of ToxinPred, these five peptides are non-toxic. The BIOPEP database gave these five peptides scores for their potential as potent DPP-IV inhibitors, according to their sequence features by the calculation of either the frequency of bioactive fragments or the potential of biological activities for the whole sequence. The result indicated that WLQL could be the most potent DPP-IV inhibitor among these five peptides, as shown in Table 2. To confirm their DPP-IV activities, these five peptides were synthesized and further evaluated for their DPP-IV inhibitory activities.

### 2.4. Protein Profiling by SDS-PAGE

The database-assisted peptide identification indicated that most peptides were derived from several major proteins such as Vitellogenin-1-like, Vitellogenin-2-like, Apolipoprotein B-100, Tesmin isoform X4, and Low-quality protein: M-phase phosphoprotein 9. The peptide amounts generated by proteases were highly correlated with the relative abundances of the original proteins. Hence, the distribution and relative abundances of SSTY proteins (before enzymatic hydrolysis) were monitored using SDS-PAGE. The SDS-PAGE showed that four main bands were observed in the gel (Figure 1B). Furthermore, these protein bands were analyzed using in-gel trypsin digestion and LC-MS/MS combined with the *P. sinensis* database. According to the result of SDS-PAGE and LC-MS/MS analysis, Vitellogenin-2-like protein with a molecular weight around 120 kDa was determined as the most intensive band, followed by the second abundant vitellogenin-1 around 70 kDa, similar to that reported by Pujiastuti et al. [4]. Vitellogenin-2-like protein matched to the highest Mascot score and sequence coverage compared to the other proteins, which suggested that it is the most abundant protein in SSTY. Vitellogenin families present in the females of nearly all oviparous species were determined as major egg yolk precursor proteins [30]. Based on the database search, the DPP-IVinhibitory peptides LPSW and VPGLAL were derived from vitellogenin-1-like protein, LPLF came from vitellogenin-2-like protein, while WLQL and LVGLPL were from Low quality proteins: M-phase phosphoprotein 9, and tesmin isoform X4 protein, respectively.

### 2.5. Determination of IC_50_ Value of Peptides against DPP-IV

**Figure A1:**
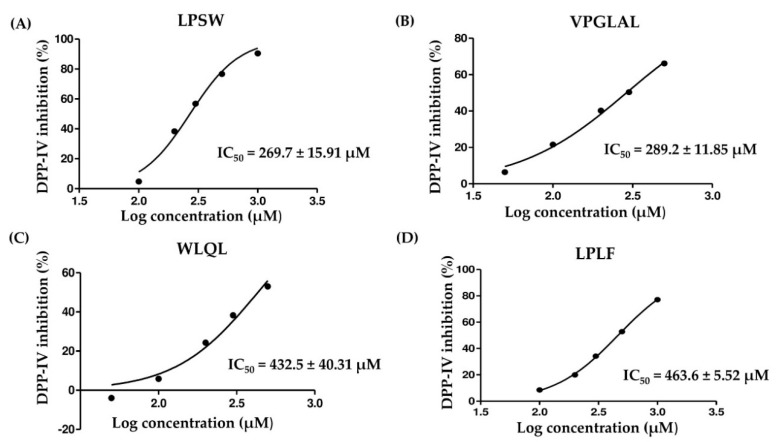
IC_50_ values of (**A**) LPSW, (**B**) VPGLAL, (**C**) WLQL, and (**D**) LPLF on DPP-IV.

LPSW, VPGLAL, LPLF, WLQL, and LVGLPL peptides from fractions of RP and SCX separations were selected for synthesis due to their high abundance and potential DPP-IV inhibition properties. Their DPP-IV IC_50_ values were determined using a nonlinear regression of inhibitory activities at five different concentrations, as shown in Figure A1. Among the selected peptides, WLQL (432.5 ± 40.31 µM) and LPLF (463.6 ± 5.52 µM) exhibited moderately high DPP-IV inhibition, while LPSW and VPGLAL displayed higher DPP-IV inhibitory activity, with DPP-IV IC_50_ values of 269.7 ± 15.91 µM and 289.2 ± 11.85 µM, respectively (Table 3). According to literature, the sequence characteristic of potent DPP-IV inhibitory peptides should possess hydrophobic amino acids (such as Ala, Gly, Ile, Leu, Phe, Pro, Met, Trp, and Val) and several hydrophilic amino acids (such as Thr, His, Gln, Ser, Lys, and Arg). Particularly, the presence of Pro/Ala is best preferred at position 2 of the N-terminus [31]. In the potent DPP-IV inhibitory sequences, the presence of hydrophilic amino acids is still unknown. However, it is known that hydrophobic amino acids may improve cooperation with the active site of DPP-IV [25]. Interestingly, among the five DPP-IV inhibitory peptides, VPGLAL, LPSW, and LPLF possessed the structure of preferred DPP-IV inhibitory substrate, that is with P residue at position 2 in the peptide, leading to their potent DPP-IV inhibitory activity, while LVGLPL had a low DPP-IV inhibitory activity (IC_50_ values >2000 µM) due to the lack of proline residue at position 2 from N-termini.

Similarly, Nongonierma et al., reported that among the 19 dipeptides with N-terminal Trp, 18 dipeptides have DPP-IV inhibitory activity, except Trp-Asp [32]. On the other hand, Lan et al., also published that a tryptophan residue was present at the N-terminal position for almost all potent peptides based on 337 dipeptides studied [33]. Besides, both authors agreed that although the DPP-IV inhibitory ability of a peptide is influenced by the residue at the N-terminal position, the C-terminal amino acids also play a role in its potency because both residues are involved in the interaction with the enzyme [31,32,33,34]. For instance, the enrichment of N-terminal Phe in dipeptides indicates strong inhibition only when combined with Ala in the C-terminal. Furthermore, N-terminal Asn shows strong inhibition when combined with His but not with Ile [33]. For the Trp-Arg-Xaa situation, the peptides showed stronger inhibitory effects than the remaining tripeptides when an acidic residue (Glu or Asp) was located at the C-terminus [34]. Additionally, large peptides containing 3 to 4 residues which have a Pro at the C terminus (Phe-Leu-Gln-Pro, Trp-Ile-Gln-Pro, Val-Leu-Gly-Pro, and Val-Arg-Gly-Pro) were also potent DPP-IV inhibitors [31].

In view of the IC_50_ values of other DPP-IV peptides derived from animals, for example chicken egg yolk (RASDPLLSV, RNDDLNYIQ, and LAPSLPGKPKPD, IC_50_ value ranged from 361.5 to 426.25 µM) [21], hen egg (ADF, MIR, and FGR, IC_50_ value were 16.83, 4.86, and 46.22 mM, respectively) [22], milk (IPSK, IC_50_ = 406.8 μM; IPPL, IC_50_ = 428.9 µM; LPLPL, IC_50_ = 325 µM) [19], camel milk (ILNKEGINY, IC_50_ = 347.8 µM; ILELA, IC_50_ = 721.1 µM) [20], and salmon gelatin (GPVA, IC_50_ = 264.7 µM; GGPASGPAV, IC_50_ = 8139.1 µM) [23], peptides derived from SSTY hydrolysates seem to have promising potent inhibitory effects on DPP-IV, especially LPSW and WLQL. The novel peptide sequences from soft shell turtle yolk hydrolysates in this study showed potent DPP-IV inhibitory properties.

BIOPEP_UWM is a reference to data about bioactive peptides that are assumed components of functional foods involved in the prevention of permanent disease [35,36]. In this study, the increasing inhibitory activity order was established as follows: LPSW > VPGLAL > LVGLPL > LPLF > WLQL based on the B value of BIOPEP-UWM (Table 2). However, the experiment results showed that LPSW had the highest DPP-IV inhibitory activity, which may be related to the formula used for calculating B value. Potential biological activity of protein fragments (B) may be calculated if peptide IC_50_ or EC_50_ (half maximal effective concentration) attributed to particular peptides is available. The program skips peptides without known IC_50_ or EC_50_ values. In other words, with a new peptide sequence, B value was just performed based on available DPP-IV inhibitory activity of peptide fragments within the peptide, instead of the whole peptide sequence. Based on the above explanation, only the prediction of DPP-IV inhibitory activity for the five peptides was possible, therefore, BIOPEP-UWM should be utilized in combination with in vitro assay to confirm the true bioactivity of peptides.

### 2.6. Stability of Peptides against DPP-IV

**Figure 4:**
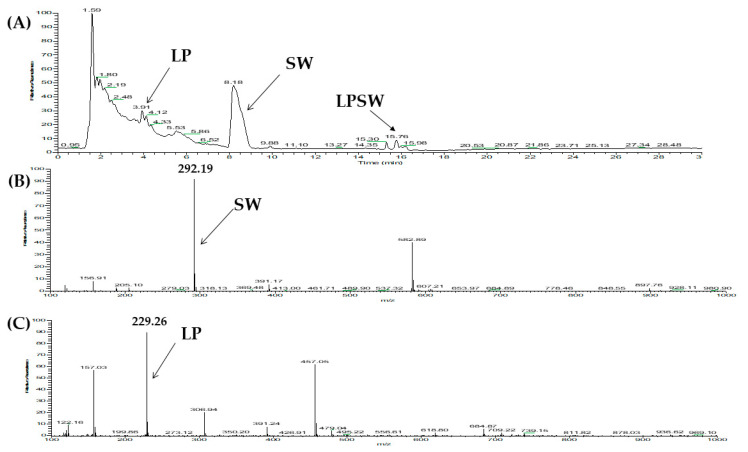
LC-MS chromatogram of (**A**) LPSW (*m*/*z* 502) pre-incubated with DPP-IV for 3 h at 37 °C. (**B**) MS spectra of SW fragment (*m*/*z* 292.12), and (**C**) LP fragment (*m*/*z* 229.25). SW and LP were fragments derived from LPSW after 3 h pre-incubation with DPP-IV.

**Figure A2:**
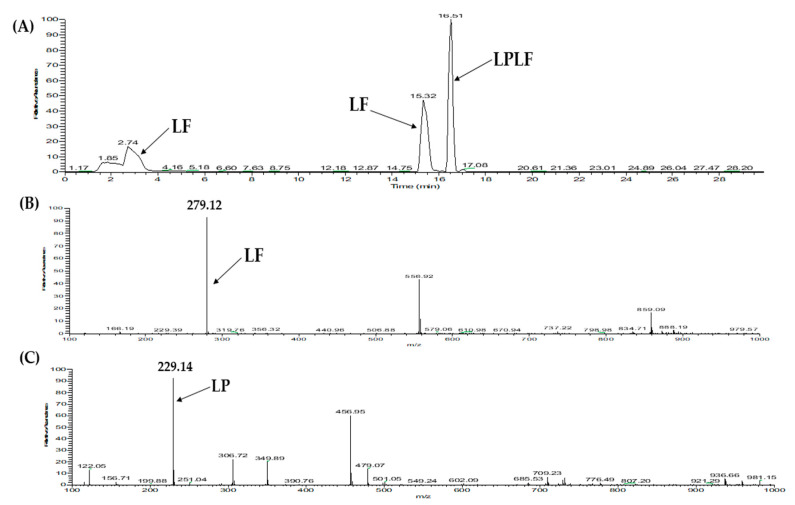
(**A**) LC-MS profile of LPLF pre-incubated with DPP-IV at 37 °C for 3 h. (**B**) MS spectrum of fragment LF (*m*/*z* 279.12), and (**C**) MS spectrum of fragment LP (*m*/*z* 229.14) derived from LPLF pre-incubated with DPP-IV at 37 °C for 3 h.

**Figure A3:**
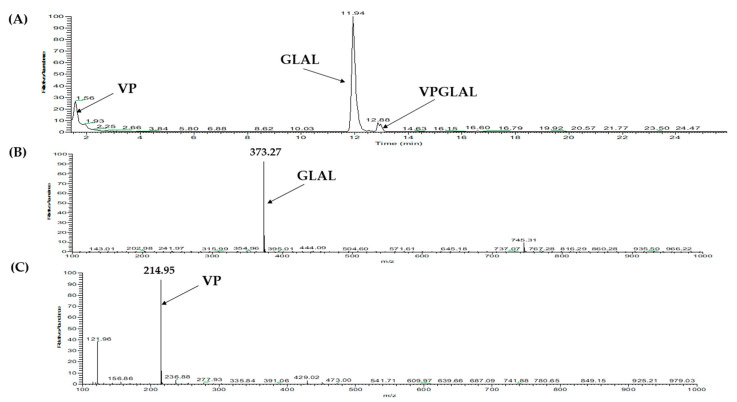
(**A**) LC-MS profile of VPGLAL pre-incubated with DPP-IV at 37 °C for 3 h. (**B**) The MS spectrum of fragment GLAL (*m*/*z* 373.27), and (**C**) the MS spectrum of fragment VP (*m*/*z* 214.95) derived from VPGLAL after pre-incubation with DPP-IV.

According to Nongierma et al., [25,37], DPP-IV inhibitory peptides can be divided into three categories depending on the result of the DPP-IV inhibitory peptide under the pre-incubation test. A true inhibitor is not affected by DPP-IV; therefore, the inhibition activity is maintained with or without the DPP-IV pre-incubation. However, a real-substrate and a prodrug are cleaved by DPP-IV, leading the inhibition activity trend to increase for prodrug-type inhibitor and to decrease for real-substrate inhibitor before and after the pre-incubated experiment. To evaluate the stability of four peptides (WLQL, LPSW, VPGLAL, and LPLF) toward DPP-IV, the pre-incubation experiment was performed, and the resulting cleavage fragment of peptide was monitored using LC-MS. According to the LC-MS analysis, WLQL was preserved after 3 h of incubation with DPP-IV at 37 °C. On the other hand, LC-MS analysis results of LPLF, VPGLAL, and LPSW pre-incubated with DPP-IV showed that they were affected by the enzyme leading to shorter fragments. Interestingly, the presentation of a Pro residue at the penultimate position, one of the characteristics of DPP-IV substrate-type inhibitors, was evident in all three peptides above. Substrate-type inhibitions normally bind to the active site where they subsequently cleave the substrate leading to smaller structures. This interaction with the active site is responsible for the inhibition of DPP-IV. As anticipated, the products of this reaction recognized as LP and SW came from LPSW (Figure 4), LP and LF were derived from LPLF (see Appendix A Figure A2), and VP and GLAL were divided from VPGLAL (see Appendix A Figure A3).

**Figure 5:**
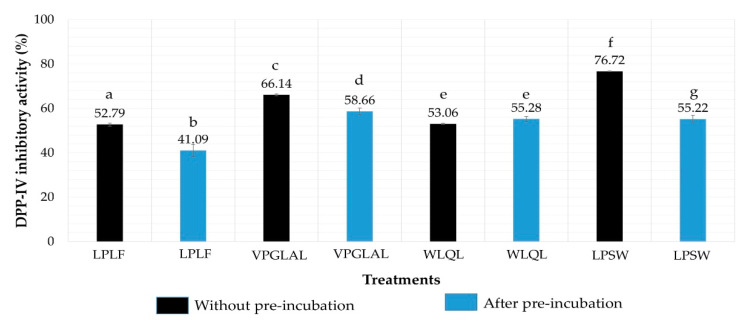
Pre-incubation experiment of LPLF, VPGLAL, WLQL, and LPSW. The error bars represent the standard deviation. Different letters (a–g) indicate significant differences between samples (*p* < 0.05). The concentration for each peptide is 500 µM.

Moreover, after incubation with DPP-IV at 37 °C for 3 h, WLQL, LPLF, VPGLAL, and LPSW were exposed to substrate Gly-Pro-p-nitroaniline (GP_pNA) to compare the inhibition activity with and without pre-incubation. Results showed that the inhibition activity (%) of WLQL (500 µM) was not changed significantly by pre-incubation with DPP-IV (from 53.06% to 55.28%). The inhibition values of the remaining peptides were decreased significantly after pre-incubation; LPLF from 52.79% to 41.09%, VPGLAL from 66.14% to 58.66%, and LPSW from 76.72% to 55.22% (Figure 5). Based on LC-MS analysis along with inhibition results of before and after pre-incubation, WLQL was found to be a true inhibitor, while LPSW, VPGLAL, and LPLF were substrates of DPP-IV. The result was also summarized in Table 3.

### 2.7. Inhibition Pattern of Synthetic Peptides

**Figure 6:**
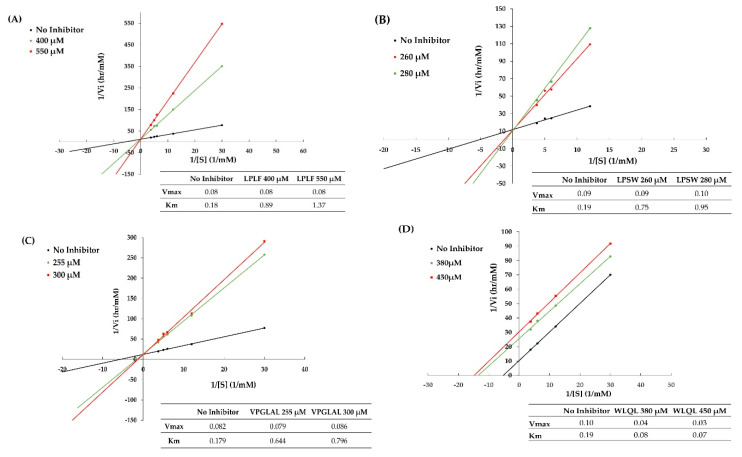
Lineweaver–Burk double reciprocal plots for dipeptidyl peptidase IV (DPP-IV) inhibition with (**A**) LPLF (competitive), (**B**) LPSW (competitive), (**C**) VPGLAL (competitive), and (**D**) WLQL (uncompetitive) were determined at concentrations corresponding to their IC_50_.

The kinetic studies of LPLF, LPSW, VPGLAL, and LVGLPL were performed using a Lineweaver–Burk plot based on several concentrations of substrate, with or without peptides. The sequences of DPP-IV inhibitory peptides that contain P or A residues at position 2 were generally observed as competitive inhibitors in most food-derived peptides [20]. As expected, the mechanism mode of LPSW, LPLF, and LVGLPL was that of competitive inhibition of the DPP-IV enzymes (Figure 6). This suggests that peptides can interact with the active site of DPP-IV and prevent the substrate binding with the enzyme. These peptides were considered as the competitive activity type because the 1/Vmax of the Lineweaver–Burk plot did not change significantly between the y-intercept for the reaction without inhibitor and the altered slope in the presence of inhibitor.

The diversity of the peptide structures can lead to the difference in DPP-IV inhibition models. The DPP-IV inhibitor also displays a noncompetitive or uncompetitive pattern. Noncompetitive type can interact with a site that is not the substrate-binding site on the enzyme and enzyme-substrate complex. Therefore, this type changes only the enzyme reaction velocity, not the organization of the substrate with the enzyme. Uncompetitive inhibition is the third kind of DPP-IV inhibitory pattern that has been suggested to carry a Trp at their N terminus leading to significant inhibition toward DPP-IV [33,34]. Similarly, based on the Lineweaver–Burk plot, WLQL belongs to the third inhibition type, an uncompetitive pattern. The inhibitor has a trend decrease Vmax and Km, which indicates WLQL can connect only to a substrate–enzyme complex and reduce the maximum enzyme activity so that it takes longer for the substrate or product to leave the active site. According to Lan et al., there are several potential advantages of an uncompetitive inhibitor such as reducing the side effects of DPP-IV inhibitors, increasing the binding affinity-mimic competitive inhibitors, and limiting the catalysis residue Ser630 in destroying the peptide bond of the peptide in the catalytic hydrolysis by DPP-IV [34]. The result of kinetics study for these five peptides was summarized on Table 3.

Additionally, the kinetic study of WLQL (IC_50_ of 432.5 ± 40.31 µM) in this study indicated uncompetitive inhibition of DPP-IV (Figure 6), which was in contrast to the competitive behavior of WL (IC_50_ value of 43.6 ± 0.9 µM) [38]. This may be due of the presence of Gln and Leu at the C terminus of WLQL. Adding two more amino acids at the C terminus changes the DPP-IV inhibitory action and the modification of targeting regions and binding sites on DPP-IV. Until now, most food protein-derived peptides were reported to be noncompetitive inhibitors or uncompetitive inhibitors [19,34,39,40,41]. Therefore, this research recommends an innovative concept for developing DPP-IV inhibitory peptides for functional food industry.

## 3. Materials and Methods

### 3.1. Material and Chemical Reagents

Chinese soft-shelled turtle eggs were collected from Pingtung City, Taiwan. Pepsin (from porcine gastric mucosa), trypsin (from bovine pancreas), α-chymotrypsin (from bovine pancreas, thermolysin (from Geobacillus stearothermophilus), human Dipeptidyl-peptidase (DPP-IV), Gly-Pro-p-nitroaniline (GP_pNA), Iodoacetamide (IAM), and linagliptin were purchased from Sigma Chemical Co. (St. Louis, MO, USA). Formic acid (FA), acetonitrile (ACN), and ammonium bicarbonate (ABC) were acquired from J.T. Baker (Phillipsburg, NJ, USA). Sequencing grade modified trypsin was purchased from Promega (Madison, WI, USA). Trifluoroacetic acid (TFA) was acquired from Alfa Aesar (Lancashire, UK), and Wang resin was obtained from Cleo Salus (Louisville, KY, USA). Deionized water (ddH_2_O) was collected using the PURELAB^®^ water purification system from ELGA LabWater (Lane End, High Wycombe, UK). All other chemicals used in this study were of reagent grades. HyperSep Retain PEP Polymeric material was purchased from Thermo Scientific Inc. Molecular weight cut-off (3 kDa MWCO) was procured from Millipore (Bedford, MA, USA).

### 3.2. Preparation of SSTY Protein Hydrolysate

SST yolk was collected after removal of whole egg white, and then the resulting yolk was lyophilized, followed by defatting using hexane before hydrolysis. Defatted yolk proteins were hydrolyzed with gastrointestinal enzymes (GI enzymes) using a two-stage protocol. The yolk protein powder (200 mg) was dispersed in 40 mL of 35 mM NaCl (adjusted to pH 2.0 using 4 M HCl) buffer and homogenized using an ultra-sonicator (Branson Digital Sonifier^®^, Fullerton, CA, USA) with 30% amplitude and the on/off pulse of 20 s/10 s for 4.20 min. Pepsin (8 mg) was added into the protein solution and the mixture was incubated at 37 °C in a thermostatically controlled shaker incubator (200 rpm). After 10 h of incubation, the pH value of the solution was adjusted from pH 2.0 to pH 8.0 with 10 M NaOH to optimize reaction conditions for trypsin and α-chymotrypsin. Trypsin (8 mg) and α-chymotrypsin (8 mg) were added into the adjusted mixture and further incubated for 10 h under the same conditions mentioned above. The resulting hydrolysate was ultra-filtrated (3 kDa MWCO membrane) by centrifugation (14,000 rpm) at 4 °C for 15 min. Moreover, the hydrolysate was desalted using the PepClean™C_18_ spin column (Thermo Scientific, Rockwood, TN, USA). Peptides with molecular weight smaller than 3 kDa were collected, lyophilized, and maintained at −20 °C for further analyses. Besides, the hydrolysis of SSTY proteins was also carried out individually with four kinds of proteases: thermolysin, pepsin, trypsin, α-chymotrypsin, and with a ratio enzyme/protein 1/25 (*w*/*w*). Based on the optimum condition of each enzyme, the enzyme digestion solutions were incubated during 16 h for thermolysin (60 °C), pepsin (37 °C), trypsin (37 °C), and α-chymotrypsin (37 °C). Reactions were performed in 25 mM ABC at pH 8.5 for all enzymes, except pepsin (35 mM NaCl at pH 2.0). After incubation times, the reactions were stopped and processed using the same method as described above.

### 3.3. Protein Profiling SSTY Protein Using SDS-PAGE

SDS-PAGE was carried out following the procedure reported by Pujiastuti et al. [4] with slight modification. The yolk proteins were purified using SDS-PAGE with 12.5% separating gel and 4% stacking gel. The molecular weight range of protein marker management (Molecular weight calibration kit, Yeastern Biotech, New Taipei City, Taiwan) for SDS-PAGE was 10–170 kDa. Before running the electrophoresis, the lyophilized yolk protein, SSTY hydrolysates derived from trypsin, pepsin, α-chymotrypsin, thermolysin, and GI enzyme (at concentration 50 µg/µL) were homogenized in 1% SDS. Electrophoresis was performed at 4 °C using a constant voltage (30 V) for approximately 30 min and then 100 V for approximately 90 min. The protein bands were recognized by staining with Coomassie brilliant blue R-250. The in-gel digestion technique was applied to identify proteins. The bands of SDS-PAGE were scattered in 100 µL of 50 mM DTT in 25 mM ABC at 37 °C for one hour. The supernatant was displaced, and proteins were alkylated using 100 µL of 100 mM IAM in 25 mM ABC for 30 min in the dark at room temperature. After replacing the supernatant, the gel was de-stained several times with 100 µL of 50% ACN in 25 mM ABC until colorless. Subsequently, the gel pieces were dehydrated using 100 µL of ACN for 5 min until shrinkage, followed by centrifugation to eliminate ACN. Afterward, the mixture was dissolved in 25 mM ABC and then sequencing grade trypsin was added at an enzyme/protein ratio of 1/20 (*w*/*w*) before being incubated at 37 °C for 16 h. The tryptic peptides were extracted from gel using 50 µL of 50% ACN in 5% TFA. The product of in-gel digestion was concentrated and kept at −20 °C before analyzing by LC-MS/MS.

### 3.4. Two Independent Bioassay-Guided Fractionations

The gastrointestinal hydrolysate (particularly smaller than 3 kDa) with the highest DPP-IV inhibition activity was fractionized with off-line SCX chromatography and reversed-phase high-performance liquid chromatography (RP-HPLC). In SCX fractionation, the mobile phase was comprised of solvent A (5% ACN and 0.2% FA in ddH_2_O) and solvent B (5% ACN, 0.2% FA, and 0.5 M NaCl in ddH_2_O). Ten fractions were collected from SSTY hydrolysate at a constant flow rate of 20 µL/min for 40 min towards each fraction. The elution buffers were applied by raising NaCl levels following sequential gradients: 0% (S1), 5% (S2), 10% (S3), 15% (S4), 20% (S5), 30% (S6), 40% (S7), 60% (S8), 80% (S9), and 100% (S10) solvent B. The fractionized products were collected individually, concentrated, and desalted (PepClean™C_18_ spin column, Thermo Scientific, Rockwood, TN, USA). Fractions were then freeze-dried and evaluated for their DPP-IV inhibition activity. Regarding HPLC fractionation, 17 fractions (R1–R17) were obtained using RP-HPLC (Hitachi Chromaster, Tokyo, Japan) with a C_18_ column (10 mm × 250 mm; particle size 5 µm, Thermo Scientific Inc., Rockwood, TN, USA). A UV detector with a wavelength at 214 nm was established for the absorbance of fractions. The mobile phase was prepared of buffer A (5% ACN and 0.1% TFA in ddH_2_O) and buffer B (95% ACN and 0.1% TFA in ddH_2_O). The gradient was processed for 100 min at a constant flow rate of 4 mL/min in the arranging for 0–60 min buffer B changing from 10% to 20%; 60–75 min from 20% to 30% B; 75–90 min from 30% to 40% B; 90–90.1 min from 40% to 80% B; 90.1–95 min isocratic elution with 80% B; 95–95.1 min from 80% B to 10% B; 95.1–100 min isocratic elution with 10% B. A fraction was collected for every peak in the chromatogram. The system was replicated various times to collect sufficient samples for further DPP-IV inhibitory assays.

### 3.5. Peptide Identification with LC–MS/MS Analysis Coupled with Database-Assisted Matching

**Figure A4:**
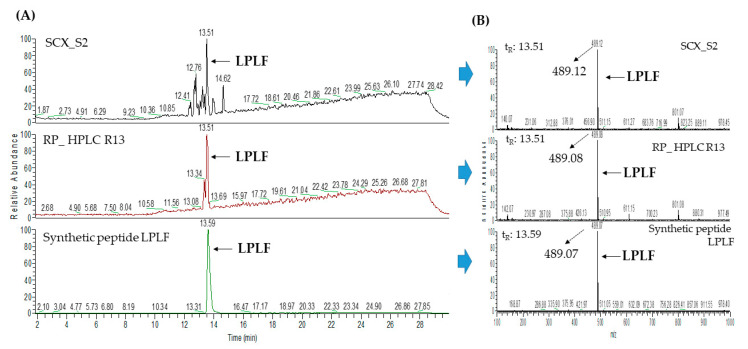
(**A**) LC–MS chromatograms of SCX S2, RP R13, and the synthetic peptide LPLF, (**B**) MS spectra of LPLF originated from fraction SCX S2 (*m*/*z* 489.12, t_R_ 13.51 min), fraction RP R13 (*m*/*z* 489.08, t_R_ 13.51) and synthetic LPLF (*m*/*z* 489.07, t_R_ 13.59).

**Figure A5:**
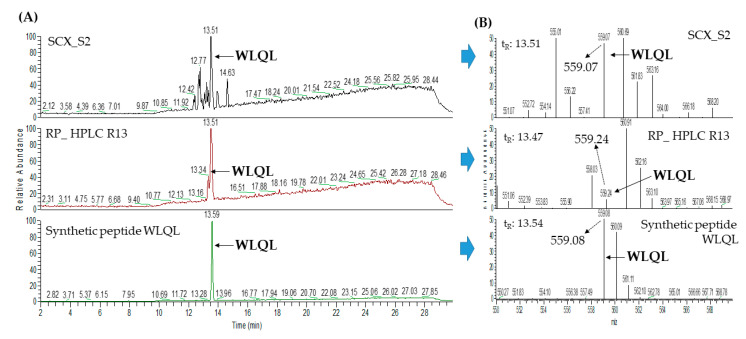
(**A**) LC–MS chromatograms of SCX S2, RP R13, and the synthetic peptide WLQL, (**B**) MS spectra of WLQL originated from fraction SCX S2 (*m*/*z* 559.07, t_R_ 13.51 min), fraction RP R13 (*m*/*z* 559.24, t_R_ 13.47) and synthetic WLQL (*m*/*z* 559.08, t_R_ 13.54).

**Figure A6:**
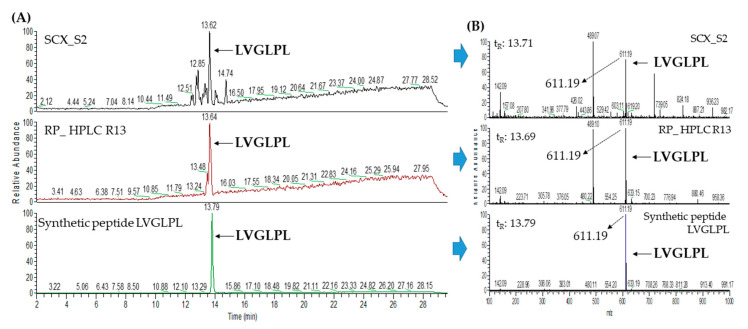
(**A**) LC–MS chromatograms of SCX S2, RP R13, and the synthetic peptide LVGLPL, (**B**) MS spectra of LVGLPL originated from fraction SCX S2 (*m*/*z* 611.19, tR 13.71 min), fraction RP R13 (*m*/*z* 611.19, tR 13.69) and synthetic LVGLPL (*m*/*z* 611.19, tR 13.79).

**Figure A7:**
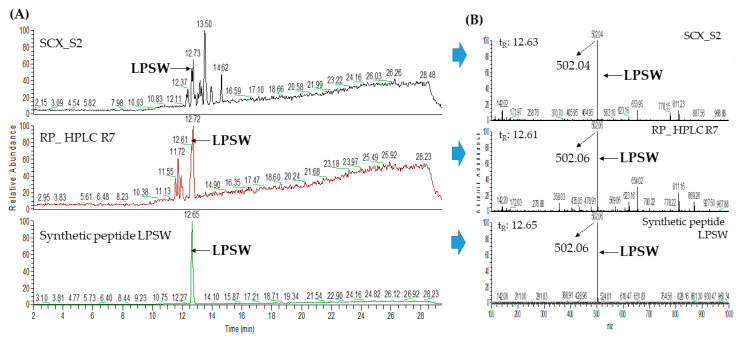
(**A**) LC–MS chromatograms of SCX S2, RP R7, and the synthetic peptide LPSW, (**B**) MS spectra of LPSW originated from fraction SCX S2 (*m*/*z* 502.04, t_R_ 12.63 min), fraction RP R7 (*m*/*z* 502.06, t_R_ 12.61) and synthetic LPSW (*m*/*z* 502.06, t_R_ 12.65).

**Figure A8:**
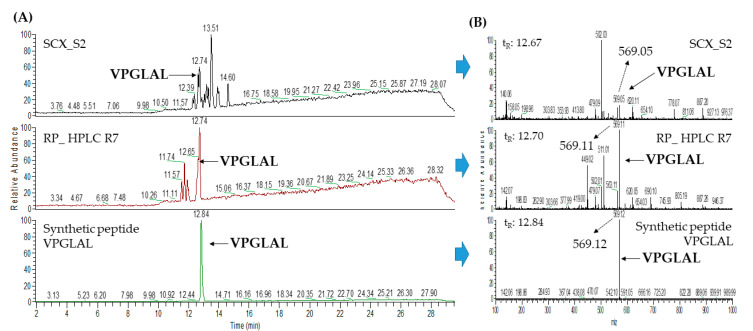
(**A**) LC–MS chromatograms of SCX S2, RP R7, and the synthetic peptide VPGLAL, (**B**) MS spectra of VPGLAL originated from fraction SCX S2 (*m*/*z* 569.05, t_R_ 12.67 min), fraction RP R7 (*m*/*z* 569.11, t_R_ 12.70) and synthetic VPGLAL (*m*/*z* 569.12, t_R_ 12.84).

Most DPP-IV inhibitory peptides derived individually from SCX and RP-HPLC were identified using the Ultimate 3000 RSLC system (Dionex, Sunnyvale, CA, USA) and analyzed with Thermo Q-Exactive™ mass spectrometer (Thermo Scientific Inc., USA). LC-electrospray ionization (ESI)-MS/MS was linked to a C_18_ column (Acclaim PepMap RSLC, 75 μm × 150 mm, Thermo Scientific). Buffer A (0.1% FA in water) and buffer B (0.1% FA in 95% ACN) were applied as the mobile phase. The elution gradient was performed in 65 min as follows: (i) sample was loaded in the first 5.5 min with 1% isocratic buffer B; (ii) in the next 39.5 min, the concentration of B buffer trend was increased from 1% to 60%; (iii) in the subsequent 10 min, buffer B level reached a peak at 80%; and finally (iv) for 10 min with 1% isocratic buffer B. The flow rate was set up at 0.25 mL/min. The tandem MS data were converted to MGF file format and investigated using PEAK Studio software (version 10.5, Bioinformatics Solutions Inc., Waterloo, ON, Canada) with PEAKS DB (database search). PEAK Studio was displayed according to the following settings (i) database: *Pelodiscus sinensis* proteins (downloaded from the NCBI database); (ii) gastrointestinal enzyme for enzyme selection; (iii) orbitrap (orbi-trap) for instrument; (iv) 50% and 0.1% for average local confidence (ALC) and MS/MS, respectively. To re-confirm all amino acid sequences, b ion and y ion values of each peptide were manually checked in the peptides’ fragmentation spectra. The sequences of five identified peptides (LPLF, WLQL, LPSW, VPGLAL, and LVGLPL) were further confirmed using synthetic peptides according to their retention times and *m*/*z* values in MS spectra (Figure A4, Figure A5, Figure A6, Figure A7 and Figure A8).

### 3.6. The Prediction of Potent DPP-IV Inhibitory Peptides Using In Silico Software

To predict the potential peptide possessing DPP-IV inhibition activity, two independent analysis tools were selected performing as BIOPEP and ToxinPred. BIOPEP (http://www.uwm.edu.pl/biochemisa/index.php/en/biopep) [35] was used anticipating the potential biological activity of the identified peptides. Another database applied to forecast toxic/non-toxic peptides was ToxinPred (http://crdd.osdd.net/raghava/toxinpred/), which is an in-silico method developed to predict and design toxic/non-toxic peptides [42].

### 3.7. Synthesis of SSTY Peptides

Potential peptides were synthesized based on the method reported by Shih et al., [28] with slight modification. Identified peptide sequences were synthesized starting from C-terminal to the end of N-terminal using Wang resins as the solid support based on the CEM Discover reactor (CEM Microwave Technology Ltd., Buckingham, UK). The process was started by preparing the incubating mixture containing Wang resin dissolved in DMF (5 mL) and the first amino acid (AA), DIC (0.5 mmol), DIPEA (1.0 mmol), and Oxyma Pure (0.5 mmol) overnight (more than 8 h). After this period, the coupling step was presented to attach the first amino acid and Wang resin using microwave irradiation (20 W) at 75 °C for 5 min. Three mL of DMF, 1 mL of acetyl anhydride, and 1 mL of 4.5% NMM were added to block the remaining hydroxyl groups of Wang resin to avoid any crosstalk reaction. The second AA was connected with the first AA by deprotection step (microwave irradiation 20 W at 75 °C for 3 min) using 20% piperidine in DMF. Afterward, the reaction was continued by combining with the pre-activated second AA (AA was dissolved in 4 mL of DMF, 1 mL of 4.5% NMM, and 0.3 mmol of HBTU), and then the coupling step was performed. The coupling–deprotection cycle was returned until last AA was completed. The completed peptide was liberated from the resin by acidic cleavage using TFA. To optimize synthesized peptide quality, TFA was further eliminated from the processing product using nitrogen flow and then desalted by the PepClean™C_18_ spin column (Thermo Scientific, Rockwood, TN, USA). The terminal product was obtained by freeze-drying concentration, and then its sequence was further verified by LC–MS/MS as described in Section 3.5. The synthetic peptide was purified using RP-HPLC with a particular gradient for each peptide to produce peptide with purity more than 95%.

### 3.8. DPP-IV Inhibitory Assay

The DPP-IV inhibitory assay was analyzed in triplicate according to the method of Lacroix and Li-Chan [43] using 96-well microplates with slight modification. Gly-Pro-p-nitroanilide was applied as a DPP-IV substrate, and the absorbance of pNA, a DPP-IV product, was recorded at 405 nm under the management of SpectraMax 190 Microplate Reader (Molecular Devices, LLC, San Jose, CA, USA). Linagliptin was used as a reference inhibitor in this experiment. All the reagents and samples were dissolved in 1% DMSO buffer. Briefly, 25 µL of 2 mM NaOH was mixed with 25 µL of 1.6 mM Gly-Pro-p-nitroanilide and preincubated at 37 °C for 10 min. Next, the sample was mixed with 25 µL of DPP-IV buffer (45 mM Tris-HCl pH 8.0; 124 mM NaCl and 2.4 mM KCl; 3 mM DTT) and incubated at 37 °C for 10 min. The blank and negative control contained 25 µL of 1% DMSO and 25 µL of DPP-IV buffer. The reaction was activated by 50 µL of DPP-IV (1 U/µL) while the blank sample used 50 µL of DPP-IV buffer instead. After 1 h at 37 °C, the mixture was terminated using 100 µL of 1M sodium acetate buffer at pH 4.0. A concentration of peptide that can inhibit fifty percentage of DPP-IV activity was defined as the IC_50_ value of DPP-IV inhibition peptide. The IC_50_ value was determined using different concentrations of inhibitor by nonlinear regression of Graphpad Prism 5.0 (GraphPad Software, Inc., La Jolla, CA, USA).

### 3.9. Determination of the Inhibition Modes of DPP-IV Inhibitory Peptides

The peptide mechanism was classified as competitive, noncompetitive, or uncompetitive inhibitory. The DPP-IV assay was carried out as mentioned in Section 3.8 with slight modification using 6 different concentrations of GP-pNA substrate (0.1, 0.2, 0.5, 1.0, 1.2, and 1.6 mM) in the absence and presence of an inhibitor (concentrations at lower and higher than IC_50_ value). The absorbance of pNA, the product of DPP-IV reaction, was recorded at 405 nm wavelength. The Lineweaver–Burk plot shows information about Km value (the affinity constant performed without inhibitor), and Vmax (the maximum rate of the reaction).

### 3.10. Stability of Synthesized DPP-IV Inhibitory Peptides

The synthetic peptide was pre-incubated with DPP-IV to determine stability against DPP-IV. Based on this peptide stability, inhibitors of DPP-IV were classified into substrate, prodrug, or true inhibitor type. According to the previous method by Nongonierma et al. [37] with slight modification, 50 μL of peptides (500 µM) dissolved in 1% DMSO were pre-incubated with 100 μL of DPP-IV (1 U/μL) in DPP-IV buffer at 37 °C for 3 h. Consequently, 75 µL of pre-incubation mixture was combined with 25 µL of 2 mM NaOH and 25 µL of 1.6 mM Gly-Pro-p-nitroanilide, and further pre-incubated at 37 °C for 10 min. The mixture was added with 25 µL of DPP-IV buffer and then incubated at 37 °C for 60 min. The reaction was ended using 100 µL of 1M sodium acetate at pH 4.0. The absorbance of each reaction was measured at 405 nm in a SpectraMax 190 Microplate Reader (Molecular Devices, LLC, San Jose, CA, USA) to determine DPP-IV inhibitory activity of peptide after a pre-incubation period. The remaining 75 µL of the original mixture was heat-inactivated at 95 °C for 10 min to end enzyme activity after which it was injected into LC-MS to confirm the stability of the peptide.

### 3.11. Statistical Analysis

The results were analyzed with ANOVA followed by Tukey’s Post Hoc test using SPSS 22.0 (SPSS Inc., Chicago, IL, USA). The confidence level of more than 95% (*p* < 0.05) has seemed a significant mean. The IC_50_ value was identified by nonlinear regression using Graphpad Prism 5.0 (GraphPad Software, Inc., La Jolla, CA, USA).

## 4. Conclusions

In this study, SSTY gastrointestinal hydrolysate showed significantly higher DPP-IV inhibition than other hydrolysates generated by the different proteases. Five novel DPP-IV inhibitory peptides, LPSW, WLQL, LPLF, VPGLAL, and LVGLPL, were identified by two independent bioassay-guided fractionations coupled with LC-MS/MS analysis and using *Pelodiscus sinensis* database. The IC_50_ value of LPSW showed the highest DPP-IV inhibitory activity (269.7 ± 15.91 µM), compared to VPGLAL, WLQL, LPLF, and LVGLPL with IC_50_ values of 289.2 ± 11.85 µM, 432.5 ± 40.31 µM, 463.6 ± 5.52 µM, and >2000 µM, respectively. Moreover, the pre-incubation experiment indicated WLQL is a true inhibitor, while LPLF, VPGLAL, and LPSW are a real-substrate type inhibitor. Furthermore, the Lineweaver–Burk plot showed that LPSW, VPGLAL, and LPLF are competitive inhibitors while WLQL is an uncompetitive inhibitor. Overall, SSTY peptides possess benefits of natural inhibitors against DPP-IV which is promising to hyperglycemia management and for the development as a functional food supplement. However, the safety level and effectiveness of these peptides need to be re-confirmed by further in vivo animal experiment.

## Figures and Tables

**Table 1:** List of the identified peptides discovered in SCX-S2, RP-R7, and RP-R13 fractions.

No	Protein	Peptide	*m*/*z*	Retention Time
Identified peptides in fraction S2 from off-line SCX				
1	Vitellogenin-1-like (gi: 558222264)	VPGLAL	569.36	30.31
2	Vitellogenin-1-like (gi: 558222264)	LPSW	502.27	29.00
3	Vitellogenin-2-like (gi: 1394701588)	LPLF	489.31	32.31
4	Tesmin isoform X4 (gi: 1394748730)	LVGLPL	611.41	35.30
5	Low quality protein: M-phase phosphoprotein 9 (gi: 1394664164)	WLQL	559.32	33.05
6	Vitellogenin-2-like (gi: 1394701588)	VLPSENPVFK	565.31	26.29
7	Vitellogenin-2-like (gi: 1394701588)	YSLY	542.26	28.84
8	Vitellogenin-1-like (gi: 558222264)	LAAF	421.24	24.73
9	Vitellogenin-1-like (gi: 558222264)	NAPLY	577.29	25.50
10	Apolipoprotein B-100 (gi: 946679552)	LNEYLEDLR	582.79	33.85
11	Apolipoprotein B-100 (gi: 946679552)	LLLY	521.33	32.25
12	Apolipoprotein B-100 (gi: 946679552)	LGLL	415.29	30.31
13	Vitellogenin-2-like (gi: 1394701588)	PISLPVGPPVPESA	680.38	36.08
14	Vitellogenin-1-like (gi: 558222264)	AQISPAPSSDF	1119.53	27.69
15	Vitellogenin-1-like (gi: 558222264)	ILDIMPAVSK	543.81	30.05
16	Vitellogenin-1-like (gi: 558222264)	MPGYAPSASDL	1108	30.00
17	Vitellogenin-1-like (gi: 558222264)	ISPAPSSDF	920.43	26.41
18	Vitellogenin-1-like (gi: 558222264)	SDDGLNF	767.32	30.39
19	Vitellogenin-2-like (gi: 1394701588)	YQIGAIE	793.41	27.97
20	Vitellogenin-1-like (gi: 558222264)	SGVGTQW	734.35	25.17
21	Vitellogenin-1-like (gi: 558222264)	ILDMPA	772.42	34.53
Identified peptides in fraction R7 from RP-HPLC				
1	Vitellogenin-1-like (gi: 558222264)	VPGLAL	569.36	30.54
2	Vitellogenin-1-like (gi: 558222264)	LPSW	502.26	29.16
3	Vitellogenin-1-like (gi: 558222264)	QELVQELHQF	635.82	27.73
4	Vitellogenin-2-like (gi: 1394701588)	SVPPELHL	446.25	26.67
5	Vitellogenin-1-like (gi: 558222264)	IRNAPLY	423.74	20.08
6	Apolipoprotein B-100 (gi: 946679552)	KIPEVTL	400.25	26.05
7	Vitellogenin-2-like (gi: 1394701588)	FADHPAIQ	449.72	20.16
Identified peptides in fraction R13 from RP-HPLC				
1	Vitellogenin-2-like (gi: 1394701588)	LPLF	489.31	33.58
2	Tesmin isoform X4 (gi:1394748730)	LVGLPL	611.41	35.50
3	Low quality protein: M-phase phosphoprotein 9 (gi:1394664164)	WLQL	559.32	33.40
4	Vitellogenin-2-like (gi: 1394701588)	LVGL	401.27	34.82
5	Vitellogenin-1-like (gi: 558222264)	PPGL	383.23	30.30
6	Vitellogenin-1-like (gi: 558222264)	QVESQLVENLR	657.85	31.63
7	Vitellogenin-1-like (gi: 558222264)	YLGDVLPGLPR	600.34	34.18
8	Pepsin A-like, partial (gi: 1394653157)	GLLGL	472.31	34.34
9	Apolipoprotein B-100 (gi: 946679552)	FLDLVIK	424.26	32.17

**Table 2:** In-silico analysis of the five overlapping peptides co-existing in both RP-HPLC and SCX fractions.

No	Co-Existing Peptides in RP-HPLC and SCX Fractionation		Length	BIOPEP		ToxinPred
				^a^ A	^b^ B	
1	RP R7 and SCX S2	VPGLAL	6	0.83	0.0004	Non-toxic
2		LPSW	4	0.75	0.0001	Non-toxic
3	RP R13 and SCX S2	LVGLPL	6	1.17	0.0008	Non-toxic
4		WLQL	4	0.50	0.0057	Non-toxic
5		LPLF	4	0.75	0.0011	Non-toxic

^a^ A: The frequency of bioactive fragment occurrence in a protein sequence, ^b^ B: Potential biological activity of protein.

**Table 3:** Summary of Dipeptidyl peptidase (DPP)-IV inhibitory peptides derived from SSTY GI protein.

Peptide Sequence	Molecular Mass (Da)	Peptide Size	DPP-IV IC_50_ (µM)	Mode of Inhibition	Peptide Type
VPGLAL	569	6	289.2 ± 11.85	Competitive	Substrate
LPSW	502	4	269.7 ± 15.91	Competitive	Substrate
LPLF	489	4	463.6 ± 5.52	Competitive	Substrate
LVGLPL	611	6	>2000	nd	nd
WLQL	559	4	432.5 ± 40.31	Uncompetitive	True inhibitor

The mode of DPP-IV inhibition was determined using the Lineweaver and Burk double reciprocal representation. nd: not determined, Peptide type was determined using pre-incubation with DPP-IV 3 h at 37 °C, and then injected into LC-MS and also compared inhibition at 500 µM of peptides with absent or present of pre-incubation with DPP-IV. nd: not determined.
